# From seq-ing to modeling: towards a molecular understanding of special properties of immature neurons in the human hippocampus

**DOI:** 10.1093/lifemedi/lnaf031

**Published:** 2025-09-30

**Authors:** Taosha Gao, Yuki Fujita, Yixing Chen, Zetian Wang, Yisha Lu, Dongchang Huang, Jingting Shen, Longying Yan, Shiyi Zhang, Yuxuan Wang, Peiming Li, Xiang Fang, Erik Hrabovszky, Chun Xu, Yijing Su, Yang Li, Lei Zhang, Yi Zhou

**Affiliations:** Institute of Neuroscience, State Key Laboratory of Neuroscience, CAS Center for Excellence in Brain Science and Intelligence Technology, Chinese Academy of Sciences, Shanghai 200031, China; Department of Anatomy and Developmental Biology, Faculty of Medicine, Shimane University, Izumo City, Shimane 693-8501, Japan; State Key Laboratory of Cell Biology, Center for Excellence in Molecular Cell Science, Shanghai Institute of Biochemistry and Cell Biology, Chinese Academy of Sciences, Shanghai 200031, China; University of the Chinese Academy of Sciences, Shanghai 200031, China; University of the Chinese Academy of Sciences, Shanghai 200031, China; State Key Laboratory of Drug Research, Shanghai Institute of Materia Medica, Chinese Academy of Sciences, Shanghai 201203, China; Institute of Neuroscience, State Key Laboratory of Neuroscience, CAS Center for Excellence in Brain Science and Intelligence Technology, Chinese Academy of Sciences, Shanghai 200031, China; Institute of Neuroscience, State Key Laboratory of Neuroscience, CAS Center for Excellence in Brain Science and Intelligence Technology, Chinese Academy of Sciences, Shanghai 200031, China; Institute of Neuroscience, State Key Laboratory of Neuroscience, CAS Center for Excellence in Brain Science and Intelligence Technology, Chinese Academy of Sciences, Shanghai 200031, China; Institute of Neuroscience, State Key Laboratory of Neuroscience, CAS Center for Excellence in Brain Science and Intelligence Technology, Chinese Academy of Sciences, Shanghai 200031, China; Institute of Neuroscience, State Key Laboratory of Neuroscience, CAS Center for Excellence in Brain Science and Intelligence Technology, Chinese Academy of Sciences, Shanghai 200031, China; Institute of Neuroscience, State Key Laboratory of Neuroscience, CAS Center for Excellence in Brain Science and Intelligence Technology, Chinese Academy of Sciences, Shanghai 200031, China; Department of Oral Medicine, School of Dental Medicine, University of Pennsylvania, Philadelphia, PA 19104, United States; Institute of Neuroscience, State Key Laboratory of Neuroscience, CAS Center for Excellence in Brain Science and Intelligence Technology, Chinese Academy of Sciences, Shanghai 200031, China; Laboratory of Reproductive Neurobiology, HUN-REN Institute of Experimental Medicine, Budapest 1083, Hungary; Institute of Neuroscience, State Key Laboratory of Neuroscience, CAS Center for Excellence in Brain Science and Intelligence Technology, Chinese Academy of Sciences, Shanghai 200031, China; University of the Chinese Academy of Sciences, Shanghai 200031, China; Department of Oral Medicine, School of Dental Medicine, University of Pennsylvania, Philadelphia, PA 19104, United States; University of the Chinese Academy of Sciences, Shanghai 200031, China; State Key Laboratory of Drug Research, Shanghai Institute of Materia Medica, Chinese Academy of Sciences, Shanghai 201203, China; State Key Laboratory of Cell Biology, Center for Excellence in Molecular Cell Science, Shanghai Institute of Biochemistry and Cell Biology, Chinese Academy of Sciences, Shanghai 200031, China; University of the Chinese Academy of Sciences, Shanghai 200031, China; Sheng Yushou Center of Cell Biology and Immunology, Department of Genetics and Developmental Science, School of Life Sciences and Biotechnology, Shanghai Jiao Tong University, Shanghai 200240, China; Institute of Neuroscience, State Key Laboratory of Neuroscience, CAS Center for Excellence in Brain Science and Intelligence Technology, Chinese Academy of Sciences, Shanghai 200031, China; University of the Chinese Academy of Sciences, Shanghai 200031, China

New neurons arising from adult neurogenesis represent a striking manifestation of neural cell plasticity that persists throughout adulthood [1]. These immature dentate granule cells (imGCs) exhibit distinct cellular and physiological properties compared to their mature counterparts and execute the function of adult neurogenesis, making them an important focus for analysis [1]. They play pivotal roles in modulating learning and memory, affective behaviors, and cognition, whereas dysregulation of imGCs has been associated with various human neurological disorders [1]. These intrinsic adult-born neurons offer potential for developing new strategies to alleviate brain disorders, stroke and injury [1]. However, our understanding of imGCs is largely based on studies involving mouse models [2]. The molecular features and regulatory mechanisms of imGCs in humans and non-human primates (NHPs) remain elusive, and their existence in adult humans was debated [2–4]. This is largely attributed to the common assumption that imGCs in different species share vast similarities. There is a lack of cross-species comparative studies and human cell-based modeling for mechanistic investigation to directly examine human imGCs. Therefore, the lack of a consistent method for identifying human and NHP imGCs and elucidating their human-specific features impedes molecular targeting and therapeutic applications of immature neurons in the human hippocampus.

**Figure 1.**
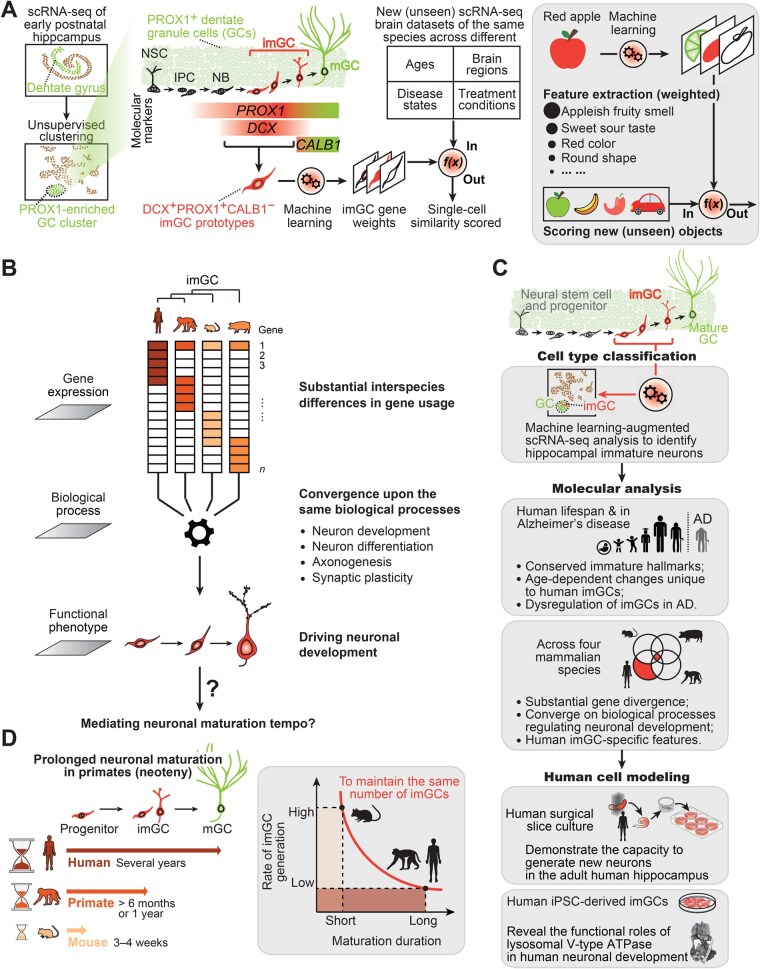
Molecular landscapes of hippocampal immature neurons across ages, species, and diseases, revealed by transcriptome-wide cell identification and human cell-based modeling. (A) Machine learning-augmented identification and molecular characteristics of immature neurons in single-cell RNA sequencing datasets of the human, macaque, and mouse hippocampus (left panel) [5, 6].This is analogous to how one might identify a red apple by its color, smell, taste, and other sensory characteristics. These features are weighted based on their importance in distinguishing a red apple from other objects. Once trained, the classifier is used to evaluate any new objects and assign a score indicating the likelihood that they are a red apple (right panel). (B) A working model based on cross-species comparison of imGCs [6]. In contrast to the traditional concept that a single genetic variant can drive cross-species cellular innovations in immature neuron regulation, their studies revealed substantial interspecies variance in highly expressed genes enriched in imGCs, which converged onto conserved biological processes, suggesting imGCs in different species may recruit and utilize species-unique molecular features to drive similar biological processes regulating neuronal development. It is intriguing for future research to establish causal roles of these human-specialized features in mediating prolonged neuronal maturation observed in primates. (C) A schematic illustration of the experimental design for the two studies conducted by the Song and Ming laboratories [5, 6]. In particular, multiple human cell-based modeling systems were developed to reveal human-specific features. First, they established a slice culture system utilizing freshly surgically resected human hippocampal tissue and showed nucleotide analog (EdU)-incorporated adult-born neuronal progenitor cells and imGCs, thereby demonstrating the capacity for neurogenesis in the adult human dentate gyrus. Second, they developed hippocampal imGC culture derived from human induced pluripotent stem cells (iPSCs) to reveal the functional role of enriched expression of v-ATPase-encoding genes in human neuronal development. (D) Neuronal maturation is prolonged in humans and other primates, a phenomenon known as neoteny [8]. An indifference curve is used to qualitatively depicting the hypothetical “imGC protracted maturation” model, which explains how low-rate, continuous neuronal progenitor generation can result in a large reservoir of imGCs [5]. New neurons are generated continuously at low frequencies but undergo protracted neuronal maturation, remaining in an immature state for a long period of time. This leads to an accumulation of a significant number of neurons with immature neuronal characteristics at any given time in the adult human hippocampus [5]. GC, dentate granule cell; imGC, immature GC; IPC, intermediate progenitor cell; mGC, mature GC; NSC, neural stem cell; NB, neuroblast; scRNA-seq, single-cell or single-nucleus RNA sequencing.

Recent efforts have utilized single-cell and single-nucleus RNA sequencing (scRNA-seq) to explore cell heterogeneity in the hippocampus of humans and NHPs (summarized in references [5–7]). However, the DCX^+^PROX1^+^ imGCs are interspersed among other cells within the dentate granule cell (GC) cluster using unsupervised clustering, even during perinatal stages [5]. Consequently, these studies have yielded inconsistent cell-typing outcomes of imGCs. Most studies have relied on prior knowledge from mouse models and have adopted study-specific criteria for cell definition, such as using various combinations of gene markers or applying transfer learning using mouse reference datasets. While these methods are effective in identifying broad cell types, they do not have the resolution to differentiate cell subtypes or substates. The Song and Ming laboratories have conducted two studies to reveal the molecular landscapes of hippocampal immature neurons across various ages, species, and diseases [5, 6]. In the first study, they utilized machine learning-augmented scRNA-seq analysis to identify human hippocampal imGCs and characterized their molecular features across the entire human lifespan and in Alzheimer’s disease [5]. In the second study, they first identified macaque hippocampal imGCs using an independent classifier [6]. They further performed cross-species comparative analyses of imGCs in four different mammalian species to reveal both conserved immature features and human-specific features that have functional roles in neuronal development [6]. Given potential species and batch variations, the authors utilized a prototype-based supervised learning approach to train classifiers tailored to each species [5, 6] (Fig. 1A). Each classifier was trained using imGC prototypes (*DCX*^+^*PROX1*^+^*CALB1*^−^ cells in the GC cluster) derived from early postnatal subjects of each species and produced a list of genes with positive and negative weights, which collectively define imGCs based on transcriptome-wide signatures, rather than relying on one or two predefined genes, such as *DCX*. The weight assigned to each gene reflects its importance in distinguishing imGCs from other cells, achieving a balance that accounts for their immature, neuronal, and regional (dentate granule) characteristics. Top positive gene weights, such as *STMN1*, were validated using histology as new markers enriched in human imGCs [5]. By assigning a score to each cell indicating its similarity to the prototype imGCs, the trained classifiers can identify immature neurons in new (unseen) scRNA-seq datasets of the brain across different ages, brain regions, and disease states within the same species [5, 6] (Fig. 1A). This approach enables the use of consistent criteria across various studies, even in cases where marker gene negativity may arise due to species-specific gene expression variations, low sequencing depth, or stochastic detection by scRNA-seq. Moreover, using mouse datasets as non-controversial “ground truth,” they benchmarked results from traditional unsupervised clustering against their machine learning approach to optimize specificity [5]. Overall, their machine learning-augment approach based on imGC prototypes from early postnatal subjects can robustly identify immature neurons in scRNA-seq datasets of the human and NHP hippocampus [5, 6].

The unique capabilities of humans relative to other species have been commonly attributed to the specialized development and function of the human brain, yet human-specific regulatory mechanisms remain largely unclear [8]. Recent cross-species studies focusing on the embryonic cortex have identified a handful of variants within individual genes or regulatory elements that contribute to the specialized cellular and physiolo­gical traits observed in humans and NHPs [8]. However, the small number of genes examined so far suggests that we have barely begun to explore the extensive and complex network of human-specific regulation [8]. In these two studies [5, 6], the authors performed cross-species analyses of hippocampal imGCs from humans, macaques, pigs, and mice and unexpectedly revealed substantial interspecies divergence at the individual gene level. For example, only 9 imGC-enriched genes (among 541 human imGC-enriched genes (1.6%)) were found shared among all four species examined. Despite such gene divergence, imGC-enriched genes of each species converge on similar biological processes involved in neuronal development. This suggests that imGCs may employ species-specific molecular signatures to maintain the immature neuronal traits (Fig. 1B). They introduced a novel concept in neuroscience proposing that, at the transcriptome level, the same cell type in different species utilizes vastly different combinations of gene machinery to drive the same biological processes. This differs fundamentally from mechanisms of cellular innovation driven by a single genetic variant, potentially enabling cellular adaptations that manifest as phenotypic traits. Moreover, the authors revealed age-related changes unique to human imGCs but not mature GCs [5]. Overall, they revealed significant species divergence, emphasizing the necessity of conducting independent molecular and functional analyses to assess adult neurogenesis in different species [5, 6].

The substantial interspecies variance observed underscores the limitations of relying solely on mouse models for investigation of human traits, highlighting the need for human cell-based models [8]. Besides utilizing post-mortem tissue for *in-situ* or immunohistology validation, these two studies employed various human cell-based models for functional analyses [5, 6] (Fig. 1C). First, a fundamental question in the field of adult neurogenesis is whether immature neurons can be born during the postnatal period. However, current methods, including scRNA-seq and histology, provide only a static snapshot and do not offer information on the birth times of cells. Given the lack of a practical approach to assess the birthdate of immature neurons in humans *in vivo*, the authors established an *ex vivo* human tissue modeling system [5]. They sliced freshly surgically resected human hippocampus from epileptic patients of varying ages and incubated slices for a brief period (1–2 weeks) in growth factor-free media containing nucleotide analog EdU, which labels newly dividing cells [5]. They observed EdU-incorporated neuronal progenitors and imGCs, directly demonstrating that the human hippocampus has intrinsic capacity to generate new neurons in adulthood [5]. Second, the authors have identified many features enriched in human imGCs, such as genes encoding Na^+^/K^+^-ATPase (*ATP1* gene family), shaker-related alpha 1 subunit of voltage-gated K^+^ channel (*KCNA* gene family), and canonical transient receptor potential cation channel (*TRPC* gene family). In particular, they identified a lysosomal vacuolar-type ATPase (v-ATPase) subtype uniquely enriched in human imGCs compared to those of macaques, pigs, and mice [6] (Fig. 1C). To investigate its functional roles, they developed *in vitro* culture models of *DCX*^+^*PROX1*^+^ hippocampal imGCs derived from two independent lines of human induced pluripotent stem cells (iPSCs) [6]. They measured the acute effects of two specific pharmacological blockers against v-ATPases at varying doses on neurite growth and neuronal activities, demonstrating the combined impact of enriched expression of v-ATPase-encoding genes in human imGCs [6]. Overall, their studies revealed distinct traits of human imGCs using human cell-based models. Future research should continue to develop such models, including longer-term cultures of human brain slices, iPSC-derived 3D modeling, and humanized mouse models [8].

A distinct cellular specialization in human neurons is their protracted maturation, which is linked to more sophisticated neuronal plasticity and connectivity impacting high-order cognitive functions [8]. Meanwhile, the lengthened maturation period inevitably increases exposure to environmental factors, thereby posing a potential risk for the onset and progression of various human brain disorders [8]. In the context of adult neurogenesis, two independent, “gold-standard” nucleotide analog (BrdU) tracing studies on macaques demonstrated that the maturation of macaques imGCs takes over 10 times longer (>6 months or a year) compared to mice (3–4 weeks) [9, 10] (Fig. 1D). By synthesizing their own data with existing research, the authors have proposed a model where new imGCs are continuously generated in the postnatal human hippocampus at low frequencies but undergo a protracted maturation process, remaining in an immature state for an extended period [5] (Fig. 1D). This leads to the accumulation of a significant number of neurons with immature characteristics in the adult human hippocampus at any given time. Given that their studies have identified numerous human-specific features that regulate imGC development, it is intriguing to further explore how these features casually mediate the delayed neuronal maturation observed in humans and NHPs [5, 6] (Fig. 1B and 1D).

Taking together, the authors have revealed the presence and molecular landscapes of hippocampal immature neurons across the human lifespan, in Alzheimer’s disease patients, and across different mammalian species [5, 6] (Fig. 1C). They identified hippocampal imGCs with transcriptome-wide immature neuronal hallmarks and revealed substantial species variance in gene enrichment and cellular dynamics. Notably, they focused on studying the unique traits of human immature neurons by developing multiple human-based modeling systems. Their studies provide the groundwork for future molecular analysis and targeting of imGCs to advance therapeutic strategies for brain diseases and facilitate our understanding of human neuronal development.
